# Hydrogen-Selective Pd-Ag-Ru Membranes and the Secret of High Permeability: The Influence of the Morphology of the Nano-Structured Coating on the Rate of Surface Processes

**DOI:** 10.3390/ijms26188765

**Published:** 2025-09-09

**Authors:** Polina Pushankina, Sergei Ivanin, Marina Papezhuk, Andranik Khachatryan, Alexander Simonov, Iliya Petriev

**Affiliations:** 1 Research Institute of Hydrogen Energy, Kuban State University, Krasnodar 350040, Russia; 2 Laboratory of Problems of Stable Isotope Spreading in Living Systems, Southern Scientific Centre of the RAS, Rostov-on-Don 344006, Russia

**Keywords:** hydrogen-permeable membranes, palladium, nanostructured coating, methanol oxidation reaction, steam reforming of alcohols

## Abstract

The efficiency of membrane reactors for steam reforming of hydrocarbons depends critically on the performance and selectivity of hydrogen-permeable membranes. In this work, a strategy for controlling the catalytic and gas-transport characteristics of Pd-Ag-Ru membranes by modifying the surface and controlling the morphology of nanostructured coatings was developed. It was found that as the process temperatures approached ~200 °C and the membrane thickness decreased, a transition to limitation of the hydrogen transfer process by surface stages was observed. Surface modification with pyramidal nanoparticles resulted in a significant increase in the hydrogen flux by up to 1.5 times compared to membranes with spiked nanoparticles and up to 2 times compared to membranes with spherical nanoparticles. The maximum difference in fluxes of up to 12 times was achieved compared to uncoated membranes. The achieved result is due to a significant increase in the active surface area associated with a systematic change in the morphology of the coatings. This aspect was a key factor in improving the catalytic activity of the material, reducing the energy barrier of sorption and accelerating the stages of hydrogen transfer through the developed membranes. Thus, modification with shape-controlled nanoparticle coatings presents an effective strategy for overcoming the limitations of the permeability of palladium-based membranes under conditions of small thickness and low temperatures. The use of the developed membranes in steam reforming reactors of alcohols can provide increased energy efficiency, conversion and purity of hydrogen.

## 1. Introduction

Currently, one of the key tasks of the modern world is the transition to environmentally friendly and sustainable energy [1,2]. In this vein, hydrogen is considered as a promising energy source for the purpose of decarbonization of the energy sector and industry [3,4]. Therefore, an important problem for its widespread use is its efficient conversion from primary raw materials [5,6]. The first place in terms of the amount of hydrogen produced is occupied by the method of steam reforming of hydrocarbons [7]. On an industrial scale, about 95% of synthesis gas is produced by steam reforming of methane [8]. The main disadvantage of this method is the relatively high reaction temperatures (about 700–900 °C) and the release of CO, which has a toxic effect on human life [9]. Conversion of bioalcohols can be considered as one of the promising methods for producing hydrogen [10,11,12,13]. Methanol and ethanol have a number of advantages as precursors for hydrogen production, since their steam conversion occurs in a relatively low temperature range (200–400 °C) and with high selectivity for hydrogen formation [14,15]. The use of bioalcohols as a raw material is a much more environmentally friendly, one might say renewable, method of hydrogen production and is characterized by a fairly low CO_2_ yield [16].

Nevertheless, traditional technologies are still widely used for reforming—large multi-tubular reactors requiring expensive and energy-intensive multi-stage hydrogen purification processes [17,18]. The purification stage in such technology is extremely important for using hydrogen in fuel cells, for which CO is a catalytic poison [19,20]. Membrane reactors are a promising replacement for traditional ones, since they allow single-stage product extraction with high selectivity [21,22,23]. The main difficulty in large-scale production of membrane reactors depends to a decisive extent on the manufacture of membrane materials capable of providing high hydrogen flows and high transfer selectivity [24]. Among various types of hydrogen-permeable membranes, dense Pd-based metallic membranes hold a special place [14,25,26]. Pd has a unique ability to reversibly sorb and diffuse atomic hydrogen, providing high permeability with almost infinite selectivity [27,28]. However, the problem of creating inexpensive, highly efficient hydrogen-permeable membranes is far from being solved at present. The reason for this is the hydrogen flux through palladium alloys in the temperature range of 200 to 300 °C and the difficulty of producing thin, but at the same time mechanically strong membranes based on them. In such a temperature range, low and unstable flows are observed, and the membranes quickly become unusable due to a high tendency to embrittlement caused by transitions between the α–β phases of palladium [29,30].

The solution to the problem of mechanical stability is alloying Pd with noble (Ag, Cu, Au, Pt) and non-noble (Ni, Y) metals [31,32,33]. This approach makes it possible to prevent the α–β phase transition and even improve the membrane permeability [34,35]. According to many theoretical and experimental studies, it has been shown that there are optimal alloy compositions capable of maintaining the membrane integrity and increasing the passing hydrogen flow [34,36,37]. One of the best known is the Pd alloy with Ag [31,38]. Thus, S. Pati et al. in their work [39] used a Pd-Ag membrane to obtain hydrogen by dry methane reforming. As a result, up to 70% of hydrogen was extracted from the reaction flow due to the high hydrogen flow through the Pd-Ag membrane. However, such a membrane is poorly resistant to sulfur and CO poisoning. The addition of Ru can reduce the rate of defect formation in the membrane, thereby preventing the leakage of impurity gases. Compared with Pd and Pd-Ag membranes, the membrane based on the Pd-Ru alloy exhibits improved resistance to poisoning by impurity gases, including H_2_S, CO and Cl-containing gases [40]. Therefore, the production of trimetallic alloys is very promising. Due to the synergistic effects, such materials combine high permeability, mechanical stability, resistance to catalytic poisons and reduced cost [41].

However, this approach does not solve the problem of deterioration of permeability with decreasing process temperatures, as in the case of alcohol reforming [14]. The deterioration of hydrogen permeability of palladium-based membranes at low temperatures is due to the kinetics of the surface stages, which slow down significantly and become the rate-limiting step for hydrogen transport in low-temperature operation. The stage of hydrogen diffusion through the Pd lattice is well described by the Arrhenius law, and its rate increases exponentially with temperature. The activation energy of diffusion is relatively small [42], and accordingly, with a decrease in temperature, diffusion still continues at a noticeable rate. Surface processes also obey the Arrhenius dependence, but with a much higher activation energy [42]. At high temperatures (>300 °C), the diffusion rate is comparatively low, and it is the rate-limiting stage, while surface processes occur very quickly. With a decrease in temperature, the rate of all processes decreases, but for processes with a high activation energy, the rate decreases much faster. As a result, at low temperatures, slow surface processes become the limiting factor due to a lack of active surface centers; accordingly, hydrogen does not have time to dissociate and be sorbed on the feed side and desorb and recombine on the permeate side, despite the fact that diffusion can still proceed quite quickly. Also, at low temperatures, impurities (CO, sulfur) can be adsorbed, poisoning the active surface centers. It can also be noted that in the literature there are practically no studies of palladium-based membranes at low temperatures. The described phenomenon is known and is mentioned in works [43,44,45]. A solution to this problem may be to increase the effective surface area of the membrane, which in turn will create a greater number of vacant sites for hydrogen sorption. Thus, W. Feng et al. [46] used hydrogen sulfide corrosion followed by its removal using oxidation-reduction reactions. This strategy made it possible to increase the surface roughness and porosity and thereby increase the hydrogen permeability of the Pd membrane by 80%. Another innovative approach is the use of nanomaterials, which have proven themselves in many areas of human activity [47,48,49,50]. The formation of a layer on the membrane surface based on metal nanoparticles, capable of sorbing hydrogen, increases the actual working surface, which leads to an increase in the number of sorption centers, the role of which is most often played by the corners and faces of crystallites [51,52]. An important feature of such particles is their structural sensitivity [53,54]. Consequently, the activity of such a catalytic coating depends to a decisive extent on the control of the shape and size of the nanoparticles and the arrangement of atoms on the surface.

Therefore, the aim of this work was to study the effect of systematic changes in the morphology of the nanostructured coating on the catalytic and gas transport characteristics of membranes based on the Pd-Ag-Ru alloy. And also the assessment of the efficiency of the developed strategy of obtaining membranes for low-temperature applications, such as alcohol reforming. Electrochemical studies in this work were used to prove the catalytic activity of the material with respect to reactions involving hydrogen. In the absence of these studies, it is difficult to prove that the increase in hydrogen flows and selectivity of the developed membranes is due to the acceleration of surface processes.

## 2. Results and Discussion

### 2.1. Gas Transport Characteristics of Pd-Ag-Ru Membranes

**Figure 1:**
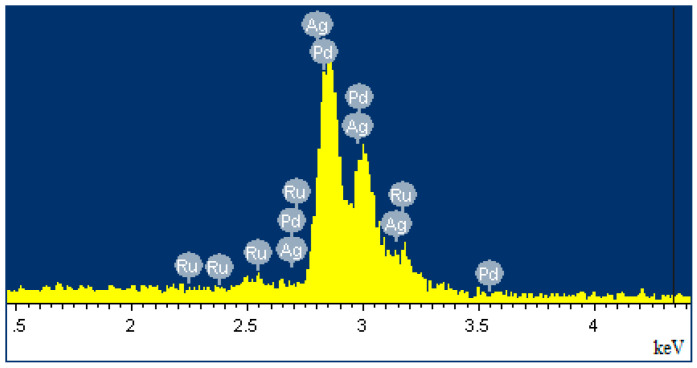
EDS spectrum of elemental composition of Pd-Ag-Ru foil samples.

In this work, Pd-Ag-Ru alloy foils were obtained, the composition of which was confirmed using EDS analysis. The results of EDS analysis, presented in Figure 1, showed a palladium content of about 74%, silver—25% and ruthenium—1%. Uniform distribution of elements was confirmed. The addition of Ru to the alloy plays an important role in improving the mechanical strength and resistance to poisoning. The atomic radius of Ru (1.3 Å) differs from the atomic radius of Pd (1.4 Å), thereby distorting and strengthening the crystal lattice of palladium. This increases the resistance of the material to deformations during the α–β phase transition in a hydrogen atmosphere and increases the service life [55]. Another important property of the alloy with ruthenium is resistance to poisoning by sulfur compounds or CO [56]. Ru creates active sites on the surface that are less sensitive to catalytic poisons, thereby increasing the service life of the membrane under reforming conditions.

**Figure 2:**
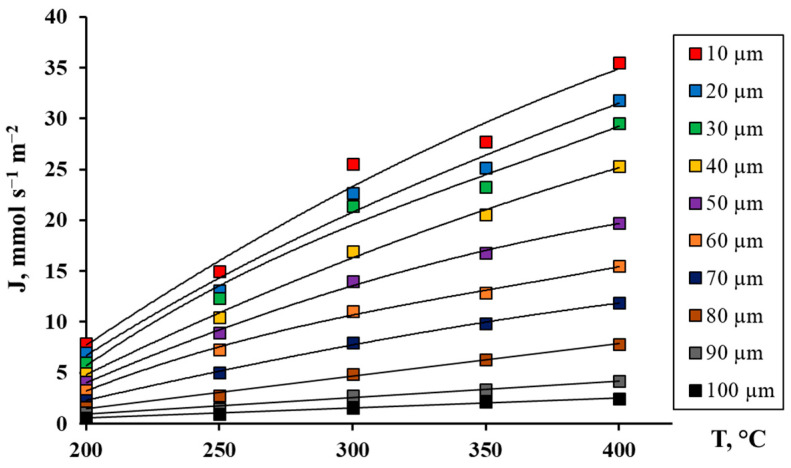
Dependence of hydrogen flow on temperature for Pd-Ag-Ru membranes of different thicknesses.

The developed Pd-Ag-Ru alloy foils of different thicknesses from 10 to 100 μm were investigated as membranes in hydrogen transfer processes in the temperature range from 200 to 400 °C. The choice of this temperature range is due to its proximity to the actual operating mode of the membranes in a steam reforming reactor of alcohols [30]. The membranes were tested for hydrogen permeability in the temperature range of 200–400 °C at an excess pressure of 0.3 MPa using the method described in Section 3.3. The obtained data, presented in Figure 2, demonstrated a clearly expressed inverse dependence of the hydrogen flux density on the membrane thickness in the entire studied temperature range. This dependence is well described by the classical equation of diffusion through a dense metal membrane (Sievert–Fick law) [57]:

(1) $$J=\frac{P}{\delta }(p_{1}^{n}-p_{2}^{n}),$$

where *J* is the penetrating hydrogen flux [mol s^–1^ m^–2^], *P* is the hydrogen permeability, [mol s^–1^ m^–1^ Pa^–0.5^], i.e., the ability of the material to pass hydrogen through its structure. The value of hydrogen permeability is directly related to the membrane thickness and allows for an effective assessment of its characteristics in the case of hydrogen transport limitation by diffusion. *δ* is the membrane thickness [m], *p*_1_ and *p*_2_ are the partial pressure of hydrogen on the retentate and permeate sides [Pa], *n* is the pressure exponent.

At the maximum temperature of 400 °C, the greatest difference in hydrogen fluxes was recorded, up to 14 times, between the membrane with a minimum thickness of 10 μm and that with the maximum thickness of 100 μm. This result is a direct consequence of the decrease in the diffusion path for hydrogen atoms in the membrane volume. This also indicates that in the region of high temperatures, hydrogen transport is limited by the diffusion stage. When approaching temperatures of ~200 °C, the nature of the dependence changes, and a reduction in the disparity in hydrogen fluxes between membranes of smaller and larger thicknesses is observed. For relatively thick membranes, the hydrogen transfer process begins to be co-limited by a combination of surface (sorption–desorption) and volume (diffusion) stages [58,59,60]. For thinner membranes near 200 °C, the process is completely limited by the sorption rate of hydrogen atoms on the feed side of the membrane and/or desorption on the permeate side [16,61,62,63]. For further studies, a membrane with a thickness of 30 μm was selected as having the most optimal strength and permeability characteristics. This thickness provides sufficient mechanical strength and resistance to deformations under the action of transmembrane pressure drop and thermal cycling, which is critical for practical use. Membranes with a thickness of 10 μm, despite high permeability, exhibit increased fragility and a tendency to damage, especially when integrated into a reactor module.

### 2.2. Morphology and Catalytic Characteristics of Nanostructured Coatings

**Figure 3:**
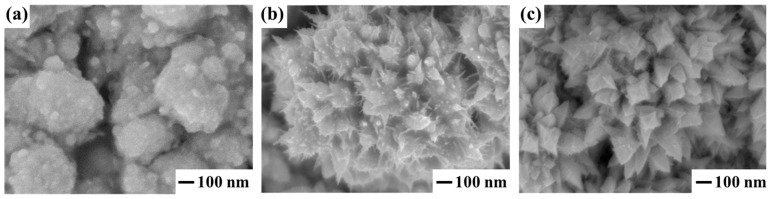
SEM images of three types of coatings consisting of spherical (**a**), spiky (**b**) and pyramidal (**c**) shaped particles.

The surface of the studied Pd-Ag-Ru foils was activated by applying a nanostructured coating based on palladium particles. This approach is aimed at imparting specific properties to the material, in particular improved catalytic activity, selectivity and hydrogen permeability, which are necessary to improve the characteristics of hydrogen-permeable membranes. The study included synthesis and comparative analysis of three different types of nanoparticles that form modifying coatings. Additional tools for adjusting the shaping (surfactant, deposition current) allowed for a systematic change in the particle shape from spherical to spiky and pyramidal. SEM images of typical representatives of each particle type are shown in Figure 3. The key aspect of the study was strict control of the dimensional parameters of the synthesized nanoparticles in the coatings. All three types of particles were obtained with a similar average size in the range of about 90–110 nm. Such strict control of the particle size in the modifying coatings was used to minimize the influence of the size factor and reliably assess the influence of the morphology factor alone on the key studied properties and characteristics of the materials.

In this work, modified Pd-Ag-Ru foils were investigated as electrodes in electrocatalytic processes. Catalytic activity and hydrogen permeability of membrane materials are inextricably linked. For both processes, the common initial stages of interaction with hydrogen are adsorption and dissociation (chemisorption). Thus, a material that easily chemisorbs hydrogen and in whose volume hydrogen atoms can quickly diffuse is often a good catalyst for hydrogenation/dehydrogenation. The high rate of hydrogen chemisorption on the palladium surface ensures abundant surface coverage by active hydrogen atoms, which immediately enter into the reaction. At the same time, the same ease of hydrogen dissolution and diffusion prevents surface poisoning, i.e., hydrogen atoms do not get stuck and do not block the active sites of the surface. This observation is important for the use of the material in a membrane reactor, where the palladium-based membrane is not just a separator, but acts as a catalyst. The reaction mixture (e.g., methanol) is fed to the feed side of the membrane, and the hydrogen formed during dehydrogenation immediately permeates the membrane and is removed from the reaction zone. Accordingly, the catalytic activity of the surface ensures the reaction, while hydrogen permeability shifts the reaction equilibrium according to Le Chatelier’s principle towards the product, increasing the conversion. This also increases selectivity, preventing secondary reactions of the obtained product with hydrogen. Therefore, the two properties synergistically enhance each other, allowing thermodynamic reactions to be carried out with a much higher yield of hydrogen. It should be noted that the dehydrogenation stage is the key stage in the methanol oxidation process, which determines the validity and importance of using these studies of hydrogen-permeable material.

**Figure 4:**
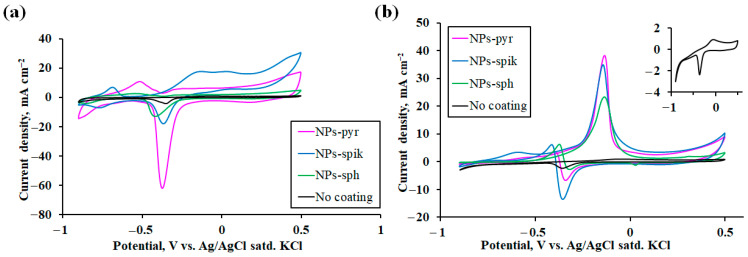
Cyclic voltammograms of Pd-Ag-Ru modified and uncoated electrodes in 1 M NaOH solution (**a**) and in the alkaline oxidation reaction of methanol (0.5 M CH_3_OH + 1 M NaOH) (**b**).

The first stage was to study the influence of the morphology of nanostructured coatings on the main characteristic of electrode materials—the electrochemically active surface area (*ECSA*). The key parameter for calculating *ECSA* was the charge integral corresponding to the peak area of the cathodic reduction in the palladium monolayer (PdO → Pd) on cyclic voltammograms (Figure 4a) [64]. The *ECSA* value was calculated using the following equation:

(2) $$ECSA=\frac{Q}{c},$$

where *Q* is the integral of the peak area of charge reduction in PdO [C], *c* is the charge required to restore the PdO monolayer [405 μC cm^−2^].

The dependence of the *ECSA* value for Pd-Ag-Ru electrodes on the morphology of nanostructured modifying coatings was experimentally established. The electrode with pyramidal nanoparticles (NPs-pyr) demonstrated the highest *ECSA* value, amounting to about 14.1 cm^2^. For the electrode with spike-shaped nanoparticles (NPs-spik), the *ECSA* value was about 4.3 cm^2^. The electrode with spherical nanoparticles (NPs-sph) was the last in the row in terms of *ECSA* value—up to 3.8 cm^2^. For the unmodified electrode, the ECSA value was 0.67 cm^2^. According to the data obtained, a statistically significant difference in the *ECSA* value is observed for electrodes modified with different types of coatings. The highest value for the electrode with NPs-pyr exceeded the value for NPs-sph by approximately 3.7 times, and for the uncoated electrode the increase was even more significant up to 21 times. This indicates a significant increase in the available active surface centers for electrochemical reactions. This effect may be due to the specific morphology of coatings with pyramidal nanoparticles containing high-index faces with an increased density of active sites [65].

It is also worth noting the difference in the anodic and cathodic peaks for NPs-pyr and NPs-spik. In the forward scan, the oxidation process (Pd → PdO) starts with the adsorption of hydroxyl groups OH^–^ on the active sites of the surface, followed by the incorporation of oxygen and the formation of an oxide layer [66]. For NPs-spik, a large number of active sites (sharp edges, vertices) facilitates the formation of oxide. Oxidation initiates more readily and proceeds faster over the entire surface due to the high density of these sites [64]. This leads to a more intense anodic current and, consequently, a higher peak. In NPs-pyr, the smooth stable faces are less prone to initiate oxidation. A higher overpotential is required to initiate oxide formation, and the process itself can proceed more slowly, which leads to a less intense anodic current and a lower peak. In the reverse scan, the process of oxide layer reduction (PdO → Pd) occurs and depends on the properties of the oxide layer itself [67]. The oxide layer formed on the high-energy surface of NPs-spik can be thicker, more heterogeneous and denser. Due to the complex morphology, the reduction process is hindered, and ions and electrons need to pass through a thicker and less conductive oxide layer. This leads to slower reduction kinetics and a less intense cathodic current. The oxide layer formed on the smooth faces of NPs-pyr is probably thinner and more homogeneous [68]. Such a layer is reduced faster to metallic palladium, the kinetics of the process are faster, which are manifested as a sharper and more intense cathodic peak.

In the second stage of the work, a comparative assessment of the electrocatalytic activity of the studied modified electrodes in the reaction of methanol oxidation in an alkaline medium was carried out. Cyclic voltammograms, typical for Pd-based materials, demonstrate the presence of two irreversible peaks in the anodic region for all electrodes (including the uncoated electrode) (Figure 4b). The peak in the forward scan is mainly due to the dehydrogenation of adsorbed methanol molecules (CH_3_OH_ads_) with the subsequent formation of adsorbed intermediates, mainly carbon monoxide (CO_ads_) on the active sites of Pd. The peak in the reverse scan is caused by the electrochemical oxidation of carbon-containing intermediates formed and accumulated on the electrode surface during the forward scan. These intermediates (especially CO_ads_) act as a catalytic poison, blocking the active sites.

The methanol oxidation reaction on Pd nanoparticles has a special profile, which is determined by the unique electronic, adsorption and catalytic properties of palladium. Thus, the surface oxide PdO is critically important on palladium. Methanol is oxidized not on pure Pd^0^, but on its oxide or at the Pd/PdO interface. Hydroxyl groups (OH^−^) from the solution are adsorbed on the oxide surface, providing oxygen for the oxidation of intermediate products [69]. As a consequence, the methanol oxidation peak on the voltammogram is observed before the oxide formation peak, which is a direct indication that the thin, pre-formed oxide layer is active. It should also be noted that it is resistant to poisoning by intermediate products (CO_ads_). The electronic structure of Pd facilitates the oxidation of CO due to the formation of active oxygen-containing centers (OH^−^). In an alkaline electrolyte (NaOH), the abundance of OH^−^ significantly accelerates the oxidation of CO_ads_ by the Langmuir–Hinshelwood mechanism [70]:

Pd–CO_ads_ + Pd–OH_ads_ → 2Pd + CO_2_ + H^+^ + e^−^

This makes Pd-based catalysts more resistant to poisoning. It is also important to take into account the extreme sensitivity of the catalytic activity of Pd to the morphology of nanoparticles, where crystallographic faces and defects play a significant role, enhancing the sorption and dissociation of methanol [71]. The studies were carried out in an alkaline medium, since it is in this medium that Pd demonstrates higher activity and stability [72]. This is due to the facilitated course of one of the key stages—CO oxidation, facilitated formation of Pd–OH_ads_ due to the high concentration of OH^−^, which is necessary for oxidative removal of poisons, as well as higher resistance to corrosion and dissolution. Thus, the special profile of methanol oxidation on Pd nanoparticles is determined by the dominant role of the surface oxide, moderate tendency to poisoning in an alkaline medium and significant sensitivity to morphology.

The obtained results can be assessed by three main parameters: (i) the anodic peak current density during forward scanning; (ii) the initial oxidation potential of methanol; (iii) the tolerance coefficient to intermediate poisoning (Table 1). The obtained experimental data revealed a significant dependence of the peak current density on the surface morphology of the studied electrodes, reflected in the following series: NPs-pyr > NPs-spik > NPs-sph >> uncoated. The peak current density values for the electrode with NPs-pyr (up to 38 mA cm^−2^) turned out to be up to 1.6 times higher than for the electrode with NPs-sph, and more than an order of magnitude higher compared to the electrode without a coating. For all electrodes the initial potential was in the negative potential region. This result indicates a decrease in the energy barrier for initiation of the methanol oxidation reaction, easier adsorption and activation of methanol molecules on the modified surface, and an increased ability to remove intermediates at early oxidation stages [73]. Accordingly, more positive values of the initial oxidation potential indicate hindered methanol adsorption and/or a strong inhibitory effect of CO_ads_, which leads to a decrease in the peak current density in a given potential region (−0.9 V–+0.5 V). The tolerance coefficient k was estimated through the ratio of the peak current density during forward (*I_f_*) and reverse (*I_b_*) scanning [74]. High values of the *I_f_/I_b_* ratio achieved for electrodes indicate efficient methanol oxidation during forward scanning, relatively low accumulation of poisoning intermediates CO_ads_ by the time of reverse scanning, as well as high efficiency of CO_ads_ oxidation during reverse scanning. This result indicates an increased resistance of the modifying nanostructured coatings to poisoning.

It is important to note the change in the magnitude of the cathode peak in an alkaline solution (1 M NaOH) and in the methanol oxidation reaction (0.5 M CH_3_OH + 1 M NaOH). The decrease in the cathode peak upon addition of methanol is a classic observation. In a pure alkaline solution, a clear pair of peaks is observed on the voltammogram:

anodic peak (corresponds to the formation of a Pd oxide film):

Pd + 2OH^−^ → PdO +2OH^−^ + 2e^−^

cathode peak (corresponds to electrochemical reduction in the oxide film):

PdO +H_2_O + 2e^−^ → Pd^0^ + 2OH^−^

The height of the most intense cathode peak is proportional to the amount of charge required to reduce the entire oxide film. When methanol is added to the solution, the picture changes. During forward scanning, the PdO oxide film is still formed. It acts as a chemical oxidizer with respect to methanol. Without waiting for electrochemical reduction, methanol molecules chemically reduce the PdO oxide to metallic Pd^0^, and they themselves are oxidized. As a result of this chemical reduction, by the time the potential during reverse scanning reaches the value of the cathode peak, the thickness of the oxide film has already decreased significantly. Thus, methanol takes some of the work from the cathode current, and less oxide remains for electrochemical reduction, which is manifested in a decrease in the cathode peak.

Thus, the increased *ECSA* of the electrodes with modified surfaces, especially with NPs-pyr, directly correlates with their improved characteristics in the methanol oxidation reaction. The special surface morphology with an increased number of active sites contributes to an increase in the active surface area and, as a consequence, to an increase in the rate of the heterogeneous electrochemical process. Also, the specific shape of the particles (sharp edges/apexes of spikes and pyramids) can contribute to a more efficient adsorption of hydrophilic hydroxyl species (OH_ads_) at negative potentials. This accelerates the CO_ads_ oxidation reaction, reducing the degree of electrode poisoning and increasing the tolerance coefficient to intermediates.

### 2.3. Gas Transport Characteristics of Modified Pd-Ag-Ru Membranes

**Figure 5:**
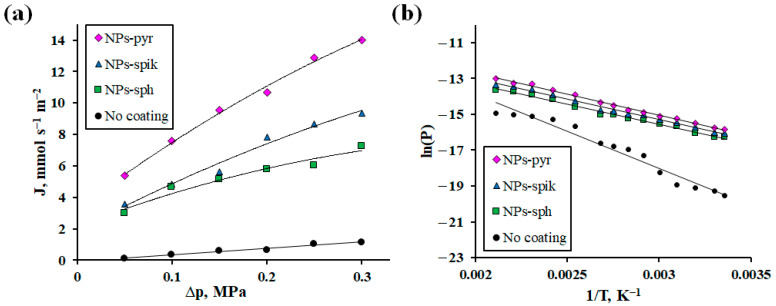
Dependence of hydrogen flow on excess pressure at 100 °C (**a**) and Arrhenius plot (**b**) for modified Pd-Ag-Ru membranes and without coating.

The obtained Pd-Ag-Ru foils with modified surface were studied in the process of hydrogen transfer as membranes. In the first series of tests, the dependence of hydrogen flux on excess pressure in the range from 0.05 to 0.3 MPa at a fixed temperature of 100 °C was investigated. As can be seen from the obtained data presented in Figure 5a, the membrane with NPs-pyr demonstrated the highest values of hydrogen flux up to 14 mmol s^−1^ m^−2^ at 0.3 MPa, which is 1.5 times higher than the values for membranes with NPs-spik and up to 2 times for membranes with NPs-sph. This result indicates a significant effect of the morphology of nanostructured coatings on the hydrogen transfer rate. The hydrogen flux values for the modified membranes turned out to be significantly higher (up to 12 times) compared to the uncoated membranes. From the obtained results, it is also possible to conclude about the effect of modification on the rate-limiting stage. For uncoated membranes, the exponent *n*, reflecting the influence of a certain rate-limiting step, was equal to 1. This corresponds to a complete limitation of transport by surface steps, which is a typical picture for Pd-based membranes in the low-temperature operating mode. In contrast, for membranes with a modified surface, values of *n* → 0.5 were recorded, which reflects a transition to a mixed limitation regime up to a significant contribution from diffusion. This shift is probably due to a decrease in the energy barrier of sorption/desorption due to a directed change in the surface morphology by forming a large number of corners, edges and defects.

The second series of tests investigated the dependence of the hydrogen flux on temperature in the range from 25 to 200 °C at a fixed overpressure of 0.3 MPa (Figure 5b). According to the data obtained, the membrane with NPs-pyr demonstrated hydrogen flux values up to 41 mmol s^−1^ m^−2^ at 200 °C, which was up to 7 times higher than the values for uncoated membranes. Calculation of the activation energy (*E_a_*) confirmed the effect of membrane surface modification and coating morphology on the rate-limiting steps of hydrogen transport. Thus, the minimum *E_a_* value was obtained for membranes with NPs-pyr (27 kJ mol^−1^), while for uncoated membranes *E_a_* was significantly higher (42 kJ mol^−1^) (Table 2). It is known from the literature that *E_a_* values below 30 indicate a significant contribution of diffusion to the hydrogen transfer process, while surface processes are characterized by significantly higher *E_a_* values [42]. The changes in the activation energy for the membranes under study are reflected by the following series: NPs-pyr < NPs-spik < NPs-sph < uncoated. Thus, the established dependence between *E_a_* and *ECSA* proves that a significant contribution to the acceleration of hydrogen transport is made not only from increase in surface roughness, but also by the creation of a special morphology of the coatings. Thus, the application of coatings with NPs-pyr, which have a high concentration of surface active centers, contributed to a decrease in the activation energy of sorption/desorption, which ensured the transition from complete limitation by the surface to predominant limitation by diffusion control.

In Table 3, the membranes manufactured in this work are compared with literature data in terms of hydrogen fluxes. The membranes manufactured in this work demonstrated very high hydrogen fluxes, compared with data from other authors, taking into account the operating conditions and layer thickness.

**Figure 6:**
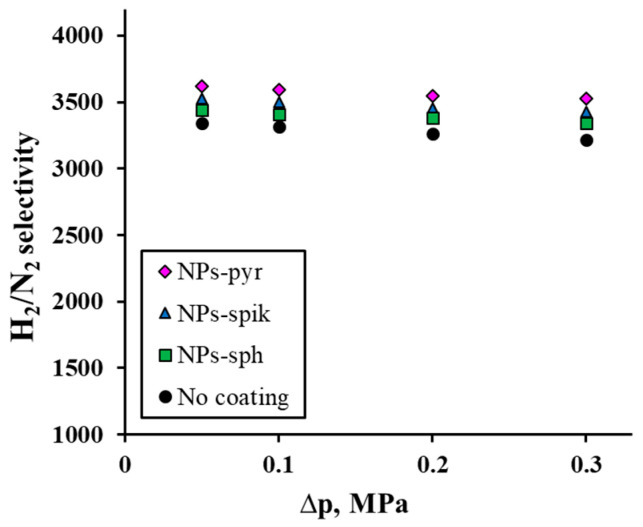
Dependence of H_2_/N_2_ selectivity on excess pressure for modified Pd-Ag-Ru membranes and without coating.

The third series of tests allowed us to evaluate the selectivity of the developed Pd-Ag-Ru membranes, determined through the H_2_/N_2_ flux ratio. The obtained data on the pressure dependence of the H_2_/N_2_ selectivity (0.1–0.3 MPa), shown in Figure 6, demonstrate a very weak inverse relationship: at the maximum pressure (0.3 MPa), the H_2_/N_2_ selectivity value decreases by only 3% relative to the values at the minimum pressure. The highest selectivity value was achieved for membranes with NPs-pyr—3621 at 0.3 MPa. An important observation is the increase in the values for membranes with a modified surface compared to the uncoated membrane. It should be noted that the dependence of the selectivity increase fully correlates with the previously described dependence of the flux density increase on pressure. This result corresponds to the model of competing mechanisms, where the dominant contribution to hydrogen transfer through the membrane is made by the dissolution–diffusion mechanism, while for nitrogen the Knudsen flux through subnanometer defects is enhanced. It should be noted that the tests were carried out in several cycles, including to determine the mechanical stability of the membranes. No hysteresis was observed during the tests, which indicates the absence of significant defects in the manufactured membranes and the adhesive strength of the modifying coatings.

## 3. Materials and Methods

### 3.1. Production of Metal Membranes Based on Pd-Ag-Ru Alloy

The basis of hydrogen-permeable membranes, thin foils of Pd_74_-Ag_25_-Ru_1_ alloy, were obtained by melting and rolling. The initial ingots of Pd, Ag and Ru metals were placed in the required percentage ratio in a copper crucible of an electric arc furnace chamber. The chamber was evacuated and filled with an inert gas—argon. During the melting process, the inverter current was increased from 20 to 120 A. Melting was carried out in several stages to achieve homogeneity and purity of the alloy. The resulting ingot of Pd-Ag-Ru alloy was subjected to multiple rolling cycles on DRM–130 rollers (Dursron, High Wycombe, UK) with intermediate annealing in a vacuum chamber to remove work hardening. As a result, foil samples of various thicknesses were obtained, ranging from 10 to 100 μm.

The elemental composition of the obtained foils was controlled by the method of energy-dispersive X-ray spectroscopy on a semiconductor energy-dispersive attachment INCA (Oxford Instruments, High Wycombe, UK) as part of a scanning electron microscope JSM-7500F (JEOL, Tokyo, Japan).

### 3.2. Surface Modification of Pd-Ag-Ru Foils

The modification of the foil surface with a nanostructured coating was carried out by the method of electrolytic deposition using a potentiostat/galvanostat R–40X (Elins, Chernogolovka, Russia) in the galvanostatic mode. The foils were pre-cleaned in an organic solvent—ethanol, followed by rinsing in deionized water. Then the samples were polarized cathodically in 0.1 M hydrochloric acid and then anodically in 0.05 M sulfuric acid at a current density of 10–20 mA cm^–2^ and a constant potential of 5 V for 30 min. This stage is necessary for a clean, chemically active and slightly rough surface, which ensures maximum adhesion, density and uniformity of the coating. Deposition was carried out in a two-electrode cell filled with an aqueous solution of tetrachloropalladium (II) acid 2% (wt.). Before deposition, the electrolyte was degassed by bubbling an inert gas—argon. A Pd foil of comparable size to the working electrode was used as a counter electrode. To control the morphology of the deposited palladium particles, a surfactant, tetrabutylammonium bromide, was introduced into the electrolyte in the following concentrations: 0 M, 0.05 M, and 0.1 M. The synthesis was carried out at a current density of 3–6 mA cm^–2^ with a stage duration of 3–5 min. To obtain spherical particles, no surfactant was added to the working solution; the synthesis was carried out at a current density of 5–6 mA cm^–2^. To obtain spiky particles, a surfactant was added to the working solution at a concentration of 0.05 M; the synthesis was carried out at a current density of 4–5 mA cm^–2^. To obtain spherical particles, a surfactant was added to the working solution at a concentration of 0.1 M; the synthesis was carried out at a current density of 3–4 mA cm^–2^. Upon completion of the process, the modified foil samples were washed with bidistilled water.

The morphology of the obtained nanostructured coatings was studied by scanning electron microscopy using a JSM–7500F device (JEOL, Tokyo, Japan) in the secondary electron detection mode.

### 3.3. Study of Gas Transportation Characteristics

The gas transport characteristics of the obtained Pd-Ag-Ru membranes were studied using a gas permeability measurement unit [82]. A membrane with a working area of 1.54 cm^2^ was mounted and fixed in the cell using a flange joint. Preparation for testing included purging the system with helium at high pressure to confirm its tightness. Before feeding hydrogen to the inlet-retentate side of the membrane, the system was evacuated. Hydrogen flows were measured in the pressure range from 0.05 to 0.3 MPa in two low- (25–200 °C) and medium-temperature (200–400 °C) intervals. The flows were monitored using mass flow meters. The selectivity of the membranes with respect to hydrogen was studied through the ratio of H_2_ and N_2_ flows in the permeate zone:

(3) $$Selectivity\;of\;\frac{H_{2}}{N_{2}}=\frac{J_{H_{2}}}{J_{N_{2}}},$$

where $J_{H_{2}}$ is the transmitted hydrogen flux normalized to the geometric area of the membrane (mol s^–1^ m^–2^), $J_{N_{2}}$ is the transmitted nitrogen flux normalized to the geometric area of the membrane (mol s^–1^ m^–2^).

### 3.4. Study of Catalytic Characteristics

The electrocatalytic characteristics of the modified and uncoated foil samples were studied using a P–45X potentiostat/galvanostat (Elins, Chernogolovka, Russia) in a standard three-electrode cell. The cell configuration included (i) a working electrode—the studied Pd-Ag-Ru foil samples, (ii) an auxiliary counter electrode—a Pt foil of comparable area, and (iii) a reference electrode—a silver chloride electrode (Ag/AgCl, saturated KCl). For a comprehensive assessment of the catalytic activity, cyclic voltammetry was used in the alkaline oxidation reaction of methanol. The electrolyte contained 0.5 M methanol and 1 M sodium hydroxide. The electrolyte was degassed before the experiments, and argon was also used for degassing. The studies were conducted in an inert argon atmosphere to remove unwanted oxygen. Multiple scanning cycles were performed in the potential range from−0.9 to +0.5 V at a scan rate of 50 mV s^−1^. The potentials were corrected for the ohmic drop determined by hardware compensation. The measured currents were normalized to the electrochemically active area of the electrode. To determine the electrochemically active surface area of the electrodes, cyclic voltammetric curves were recorded in a background electrolyte (1 M sodium hydroxide) in the same range of potentials and scan rates.

## 4. Conclusions

The conducted study revealed the effect of applying a nanostructured modifying coating and its morphology on the functional characteristics of hydrogen-permeable Pd-Ag-Ru membranes. The study of the dependence of the flux on the membrane thickness made it possible to clearly trace the transition from limiting hydrogen transport by diffusion to limiting it by surface processes (sorption/desorption). To remove surface limitations, Pd-Ag-Ru membranes were modified with a coating with different morphology of nanoparticles. Electrochemical studies of the modified samples confirmed the hypothesis about the effect of the morphology of the coatings on their functional characteristics. Thus, the highest peak current density in the methanol oxidation reaction was demonstrated by samples with pyramidal nanoparticles—up to 38 mA cm^−2^. The obtained values were up to 1.6 times higher than for samples with spherical nanoparticles and an order of magnitude higher than for uncoated samples. These data fully correlated with the data on the gas-transport characteristics of the membranes studied in the processes of hydrogen transport. Thus, for membranes with pyramidal nanoparticles, the values of hydrogen flux density were recorded up to 1.5 times higher compared to membranes with spiked nanoparticles and up to 2 times higher compared to membranes with spherical nanoparticles. The maximum difference in flows of up to 12 times was achieved compared to uncoated membranes. The achieved result is due to a significant increase in the active surface area associated with a systematic alteration in the surface morphology. This aspect was a key factor in improving the catalytic activity of the material and accelerating the stages of hydrogen transfer through the developed membranes. An additional confirmation of the acceleration of surface processes was the recorded decrease in the energy barrier of sorption. Thus, modification with shape-controlled nanoparticle coatings presents an effective strategy to overcome the permeability limitations of palladium-based membranes under conditions of small thickness and low temperatures. The use of the developed membranes in steam reforming reactors of alcohols can provide increased energy efficiency, conversion and purity of hydrogen.

## Figures and Tables

**Table 1:** Statistical parameters obtained as a result of cyclic voltammetry of the studied electrodes.

Electrodes	*I_f_*, mA cm^−2^	E_onset_, V	*I_b_*, mA cm^−2^	*I_f_*/*I_b_*
NPs-pyr	38.3	−0.67	3.7	10.4
NPs-spike	34.7	−0.71	6.1	5.7
NPs-sph	23.4	−0.51	6.0	3.9
No coating	0.92	−0.63	0.17	5.4

**Table 2:** Calculation parameters and activation energy values for the developed membranes.

Membrane	Ea, kJ mol^−1^	Temperature Range, °C	∆p, MPa
Pd-Ag-Ru/NPs-pyr	42	25–200	0.3
Pd-Ag-Ru/NPs-spik	30	25–200	0.3
Pd-Ag-Ru/NPs-sph	28	25–200	0.3
Pd-Ag-Ru	27	25–200	0.3

**Table 3:** Comparison of hydrogen fluxes of Pd-based membranes.

Membrane	Support	Thickness of Selective Layer, µm	Temperature, °C	Pressure, MPa	H_2_, Flux, mol m^−2^ s^−1^	Reference
Pd	Al_2_O_3_	4	400	0.02	0.04	[75]
Pd-Au	PSS	7	350	0.1	0.881 × 10^–2^	[76]
Pd-Cu	Al_2_O_3_	4	340	0.1	0.028	[77]
Pd-Cu	PSS	23.74	175	0.1	0.007	[78]
Pd-Cu	Al_2_O_3_	3.5	100	0.5	0.06	[79]
Pd-Ag	–	25	200	0.006	0.005	[80]
Pd-Ag	–	100	200	0.1	0.049	[81]
Pd-Ag-Ru modified	–	30	200	0.3	0.04	this work
Pd-Ag-Ru	–	30	200	0.3	0.006	this work

## Data Availability

The data presented in this study are available on request from the corresponding author.
